# Enhanced reverse zoonotic potential and immune evasion by omicron JN.1 variant

**DOI:** 10.1016/j.isci.2025.112824

**Published:** 2025-06-06

**Authors:** Jiaxin Hu, Fuwen Zan, Yixin He, Xiuyuan Ou, Xiaolu Tang, Yan Liu, Xing Lu, Pei Li, Zhixia Mu, Siwen Dong, Yahan Chen, Lin Tan, Mengmeng Cao, Pinghuang Liu, Terrence Tsz-Tai Yuen, Jian Lu, Zhaohui Qian

**Affiliations:** 1 NHC Key Laboratory of Systems Biology of Pathogens, Institute of Pathogen Biology, Chinese Academy of Medical Sciences & Peking Union Medical College, Beijing, China; 2 MOE Key Laboratory of Pathogen Infection Prevention and Control, Peking Union Medical College, Beijing, China; 3 State Key Laboratory of Respiratory Health and Multimorbidity, Chinese Academy of Medical Sciences & Peking Union Medical College, Beijing, China; 4 Department of Microbiology, School of Clinical Medicine, Li Ka Shing Faculty of Medicine, The University of Hong Kong, Pokfulam, Hong Kong, China; 5 College of Life Sciences, Peking University, Beijing, China; 6 Key Laboratory of Animal Epidemiology of the Ministry of Agriculture, College of Veterinary Medicine, China Agricultural University, Beijing, China

**Keywords:** Immunology, Virology

## Abstract

SARS-CoV-2 infects not only humans but also animals, posing reverse zoonotic risks. As SARS-CoV-2 rapidly evolves, JN.1 has become dominant globally. In this study, we determined the susceptibility of XBB.1.16, EG.5.1, BA.2.86, and JN.1 to 27 different animal angiotensin-converting enzyme 2 (ACE2) orthologs using pseudoviruses, and found that JN.1 displayed substantially higher overall reverse zoonotic risk potential compared to other variants except for EG.5.1. Live virus infection experiments further confirmed higher infectivity of JN.1 than BA.2.86. Mechanistic analyses revealed that L455S might be responsible for substantial increase in overall fusogenecity and infectivity by lowering S protein thermostability. Additionally, we also found that L455S mutation enhanced immune evasion of SARS-CoV-2, and XBB breakthrough infection increased levels of neutralization antibodies against JN.1. Together, our findings offer a better mechanistic understanding of CoV entry, host range, evolution, and immunogenicity and highlight the importance of surveillance of susceptible hosts to prevent potential outbreaks.

## Highlights

• JN.1 exhibits a higher potential for reverse zoonotic transmission risk compared to BA.2.86
• The L455S mutation decreases receptor binding while increasing fusogenicity and infectivity
• Mechanistically, the L455S mutation enhances viral infectivity by reducing S protein stability
• Breakthrough infection with XBB strengthens protection against JN.1 infection

## Graphical abstract

**Figure:**
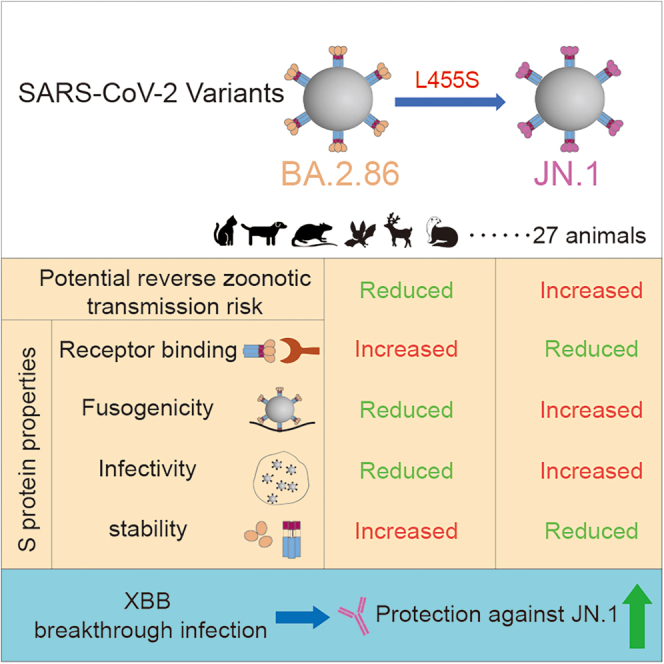

## Introduction

Coronavirus disease 2019 (COVID-19) is caused by a newly emerged coronavirus, severe acute respiratory syndrome coronavirus 2 (SARS-CoV-2).^1^^,^^2^^,^^3^ As of April 30th, 2024, there are at least over 775 million confirmed cases and over 7 million reported deaths worldwide.^4^ SARS-CoV-2 is a plus-stranded RNA virus and classified as a member of *sarbecovirus* subgenus in *beta-coronavirus* genus.^5^ Due to its nature as an RNA virus, it inherently undergoes mutations and recombination, leading to continuous emergence of new variants that cause one and another infection waves.^6^^,^^7^^,^^8^

The spike (S) protein of SARS-CoV-2 is responsible for virus entry, tissue tropism, and host range. S protein is a trimer and belongs to class I viral fusion protein.^9^ Each monomer contains two subunits, S1 and S2, separated by a furin cleavage site.^2^^,^^10^ S1 contains the receptor binding domain (RBD) and is responsible for binding to its receptor, human angiotensin-converting enzyme 2 (hACE2),^2^^,^^10^^,^^11^ whereas S2 possesses all membrane fusion machinery. Proteolytic cleavage at S2′ or nearby is required for activating membrane fusion potential of S2 and achieving viral and cellular membrane fusion.^12^^,^^13^

**Figure 1:**
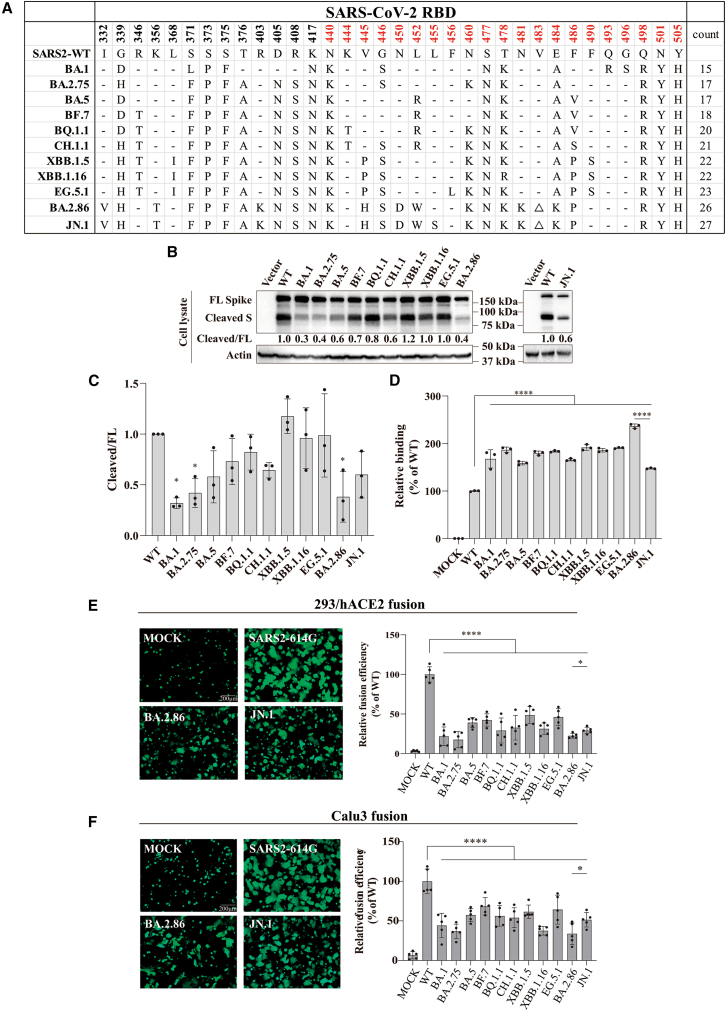
Receptor binding and fusogenicity of S proteins of omicron variants (A) Amino Acid sequence alignment of RBDs from different omicron variants. Dashed lines represent residues identical to WT. Residues of RBM are shown in red. (B and C) Cleavage of S proteins from different omicron variants. Plasmids encoding codon-optimized S proteins were transfected into HEK293T cells and the cells were then lysed at 40 h post-transfection. The expression of S proteins was detected by western blot using rabbit polyclonal anti-S2 antibodies. Actin served as the loading control. Experiments were done three times and one representative was shown. Band densities were quantified using ImageLab software to determine the cleaved/FL ratio and normalized to WT. FL S, full-length spike protein. (D) Binding of soluble hACE2 to S proteins of omicron variants. HEK293T cells transiently expressing the indicated S proteins were incubated with biotinylated hACE2 followed by Alexa Fluor 488-conjugated streptavidin and then analyzed by flow cytometry. Experiments were done three times, and one representative was shown. (E and F) Cell-cell fusion mediated by S proteins of omicron variants. HEK293T cells were co-transfected with indicated S protein and eGFP. The cells were detached with trypsin at 48h post-transfection and overlaid on the HEK293 cells expressing hACE2 or Calu3 cells. After 2 h of incubation, five randomly selected images of syncytia were captured using a fluorescent microscope. Fusion areas were quantified using ImageJ software and normalized to WT as fusion efficiency. Experiments were done three times in triplicate and one representative is shown. The statistical difference relative to WT was determined by one-way ANOVA with Dunnett’s multiple testing correction (*n* = 3 for (C) and (D); *n* = 5 for (E) and (F)). The statistical difference between BA.2.86 and JN.1 was determined by a two-tailed t-test (*n* = 3 for (C) and (D); *n* = 5 for (E) and (F)). Error bars indicate SEM. ∗*p* < 0.05, ∗∗*p* < 0.01, ∗∗∗*p* < 0.001, ∗∗∗∗*p* < 0.0001. Scale bar = 200μm. See also Figure S1.

The omicron variant (B.1.1.529), initially identified on November 24, 2021,^14^ gained attention for its extensive mutations in the spike (S) protein, particularly in RBD, resulting in notable immune evasion along with changes in virological characteristics.^15^^,^^16^^,^^17^ Subsequent omicron variants, like BQ.1.1, XBB, BA.2.86, JN.1, and so on exhibiting even more mutations (Figure 1A), continue to pose threats to global public health by displaying stronger immune evasion.^18^^,^^19^^,^^20^^,^^21^

SARS-CoV-2 is thought to have emerged from bats, likely through an intermediate animal host.^22^^,^^23^^,^^24^^,^^25^ Recently, we and others showed original SARS-CoV-2 Wuhan strain and early omicron variants like BA.1 might have broad host range.^23^^,^^26^^,^^27^^,^^28^^,^^29^^,^^30^ Many animals including ferrets, cats, minks, tigers, hamsters, white-tailed deer, and dogs to a lesser degree, are documented for infection of SARS-CoV-2.^26^^,^^28^^,^^31^^,^^32^^,^^33^^,^^34^ Outbreaks of SARS-CoV-2 have been reported in several mink farms in Europe, and the viruses were transmitted back and forth between humans and minks repeatedly.^35^^,^^36^^,^^37^ Of note, a significant portion of free-ranging white-tailed deer in the United States were also detected positive for SARS-CoV-2, which might serve as an additional wildlife reservoir for SARS-CoV-2.^38^ The adaptation of SARS-CoV-2 in animals might lead to changes in viral pathogenesis and transmission among humans and bring more challenges for future pandemic control.

Currently, JN.1 has become the dominant variant circulating the world. Compared to the original Wuhan strain, there are 27 amino acid changes found in JN.1 RBD, and several of them make direct contact with hACE2,^39^^,^^40^^,^^41^ which might affect its affinity with human and animal ACE2s and change the potential animal host range. In this study, we systematically compared the characteristics of S proteins of different omicron variants including JN.1, regarding S protein expression, cleavage, receptor binding, fusogenicity, and pseudoviral transduction. Moreover, we determined the susceptibility of XBB.1.16, EG.5.1, BA.2.86, and JN.1 to 27 different animal ACE2 orthologs, revealing potential new reverse zoonotic risks, and also identified residue 455 critical for increase of membrane fusion and infectivity. Finally, we also showed that XBB breakthrough infection enhanced humoral immunity against JN.1 infection.

## Results

### Virological characteristics of omicron variants

A single mutation might significantly impact the properties of the S protein, leading to changes in its biological features. For instance, the D614G mutation results in more open conformation of S protein, enhancing its affinity with hACE2.^42^^,^^43^ With multiple mutations accumulating in S proteins of various omicron variants (Figure 1A), how might these changes influence the characteristics of S protein? To address this, we obtained S protein expressing constructs of 11 different omicron variants, including JN.1. These plasmids were transfected into HEK293T cells and the expression levels were determined using polyclonal anti-S2 antibodies by western blot analysis, with β-actin serving as a loading control. While the full length (FL) S proteins of most omicron variants were expressed at levels similar to the wild-type (WT) control (SARS-CoV-2-G614) (Figures 1B and 1C), the cleaved S varied significantly, with BA.1 (0.3), BA.2.75 (0.4), and BA.2.86 (0.4) displaying substantially lower cleaved/FL ratios than WT control (1.0).

Next, we measured the affinity of the S proteins of various omicron variants with hACE2. The S protein expressing HEK293T cells were incubated with soluble hACE2, and their binding affinities were determined by flow cytometry, adjusting for the levels of S proteins present on the cell surface (Figure S1). Consistent with the previous reports,^41^^,^^44^^,^^45^^,^^46^ all omicron S proteins exhibited marked increases in affinity with hACE2, ranging from 147% to approximately 240% of the WT S protein (Figure 1D). Of note, BA.2.86 displayed the highest affinity with hACE2, suggesting a potential contribution to its increased transmission of BA.2.86 (Figure 1D). Strikingly, JN.1 exhibited only 147% binding of WT and substantially lower than BA.2.86, indicating that L455S mutations might negatively affect receptor binding, in agreement with previous reports.^47^^,^^48^

A cell-cell fusion assay was also conducted to compare the ability of different omicron variants to mediate membrane fusion. All Omicron S proteins exhibited significantly reduced syncytium formation in both 293/hACE2 and Calu3 cells (Figures 1E and 1F), compared to WT S protein. BA.2.86 only exhibited about 23% and 34% fusogenicity of WT S protein in 293/ACE2 and Calu3 cells, respectively, in spite of displaying over 2-fold increase in receptor affinity, while JN.1 displaying marked lower binding affinity to hACE2 than BA.2.86 induced substantially more syncytium than BA.2.86 in both cells (29% vs. 23% for 293/hACE2 and 51% vs. 34% for Calu3) (Figures 1E and 1F).

**Figure 2:**
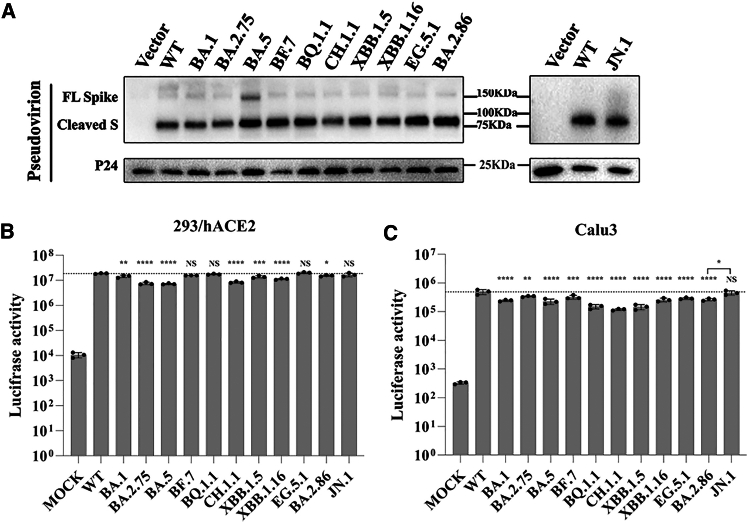
Transduction of various omicron S pseudovirions on 293/hACE2 and Calu3 cells (A) S proteins incorporation in pseudovirions. Pseudovirions with different omicron S proteins were pelleted down by centrifugation through 20% sucrose cushion and separated in a 10% SDS-PAGE. Detection of S proteins in pseudovirions was performed by Western blot using rabbit polyclonal anti-S2 antibodies. The p24 served as the loading controls. Experiments were done three times and one representative was shown. (B and C) Entry of pseudovirions of omicron variants. HEK 293/hACE2 and Calu3 cells were transduced with omicron S pseudovirions and lysed at 40 h post-transduction. The transduction efficiencies were determined according to luciferase activities. Experiments were done three times in triplicate and one representative is shown. The statistical difference relative to WT was determined by one-way ANOVA with Dunnett’s multiple testing correction (*n* = 3). The statistical difference between BA.2.86 and JN.1 was determined by a two-tailed t-test (*n* = 3). Error bars indicate SEM. ∗*p* < 0.05, ∗∗*p* < 0.01, ∗∗∗*p* < 0.001, ∗∗∗∗*p* < 0.0001.

Furthermore, we also compared entry efficiencies of omicron variants into both 293/hACE2 and Calu3 cells using an S protein pseudotyped lentivirus system. All omicron S proteins were well incorporated into pseudovirions (Figure 2A) with cleaved S in majority, which exhibited infection at levels similar to, or slightly less than, WT S pseudoviruses (Figures 2B and 2C). BA.5 exhibited the lowest level of infectivity in 293/hACE2, while CH.1.1 was the lowest in calu3. Of note, JN.1 showed higher infectivity than BA.2.86 in both 293/hACE2 and Calu3 cells (Figures 2B and 2C), consistent with the increases in fusogenicity (Figures 1E and 1F), indicating that, besides strong immune evasion, higher infectivity might also contribute to the quick rise of JN.1 in circulation. In summary, our data reveal increased binding affinity, attenuated fusogenicity, and slightly lower transduction efficiency of S proteins of omicron variants compared to the WT S protein.

### JN.1 variant exhibited higher susceptibility with various animal ACE2 orthologs

**Figure 3:**
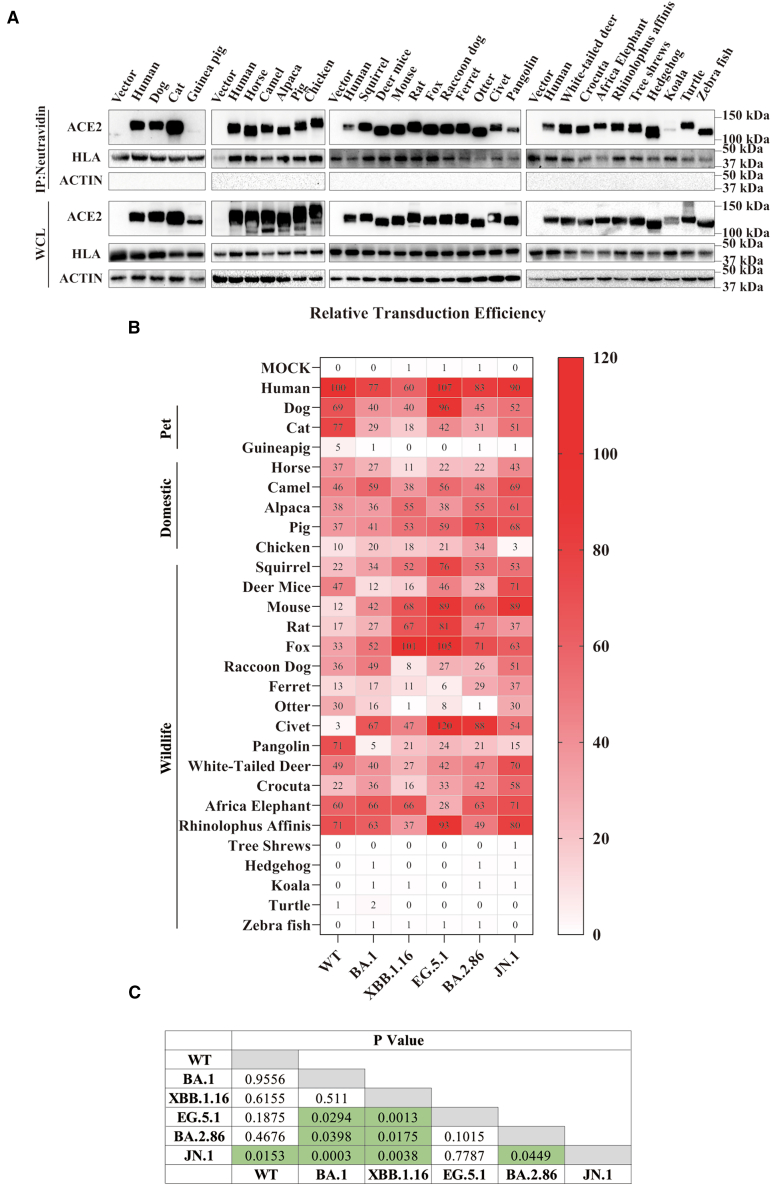
Entry of different omicron pseudovirions on HEK293 cells expressing various animal ACE2s (A) Analysis of expression of various ACE2s on HEK293 cell surface by cell surface biotinylation assay. HEK293 cells transiently expressing different FLAG-tagged different ACE2s were labeled with EZ-link Sulfo-NHS-LC-LC-biotin on ice, and lysed with RIPA buffer. Biotinylated proteins were enriched with NeutraAvidin beads and detected by Western blot using mouse monoclonal anti-FLAG M2 antibody. Human Leukocyte Antigen (HLA) and actin served as controls. WCL, whole cell lysate. (B) Relative transduction efficiencies of different omicron variant pseudovirions on HEK 293 cells expressing various ACE2 orthologs. HEK293 cells transiently expressing different animal ACE2s were transduced with pseudovirions of WT, BA.1, XBB.1.16, EG.5.1, BA.2.86 and JN.1. Relative transduction efficiencies were normalized to WT/hACE2(100%). Experiments were performed three times in triplicate, and the averages are shown as the heatmap. (C) Statistic analyses of differences of transduction efficiencies between different omicron variant pseudovirion by Wilcoxon test. Significant differences indicated by *p* < 0.05 are highlighted in green background. See also Figure S2.

Previous studies have demonstrated that early variants of SARS-CoV-2 could use various animal ACE2s as entry receptors,^23^^,^^49^^,^^50^^,^^51^ raising concerns about potential reverse-zoonotic transmission. Newly emerged omicron variants like BA.2.86 and JN.1 display additional multiple mutations in RBM, which might influence the host susceptibility among animals. To evaluate this possibility, ACE2s from 27 animals (Figure S2) were chosen, representing pets (dog, cat, guinea pig, and zebrafish), domestic animals (horse, camel, alpaca, pig, and chicken), and wildlife animals (squirrel, deer mice, mouse, rat, fox, raccoon dog, ferret, otter, civet, pangolin, white-tailed deer, Crocuta, African elephant, *Rhinolophus affinis* bat, tree shrew, hedgehog, koala, and turtle). A phylogenetic tree based on the amino acid sequences of these ACE2 proteins was constructed and 20 residues of these animal ACE2 orthologs potentially making direct contact with the RBD were listed in Figure S2. The plasmids encoding flag-tagged individual animal ACE2s were transfected into HEK293 cells, and their expression on HEK293 cells surface was determined by surface biotinylation assay. Most of animal ACE2s were expressed and present on cell surface at levels similar to hACE2, except for guinea pig and koala (Figure 3A), in agreement with previous reports.^23^^,^^28^

To determine the susceptibility of animal ACE2 orthologs to infection of omicron variants, ACE2 expressing cells were transduced with various S protein pseudoviruses. The relative infectivity was calculated based on transduction efficiency of WT pseudoviruses on hACE2 cells (WT/hACE2), set as 100%. In agreement with previous reports,^26^^,^^27^^,^^28^^,^^31^ WT S pseudoviruses showed relative broad potential host range (Figure 3B). Eight ACE2 orthologs, including dog (69%), cat (77%), camel (46%), deer mice (47%), pangolin (71%), white-tailed deer (49%), African elephant (60%), and *Rhinolophus affinis* bat (71%), exhibited more than 40% of WT S/hACE2 infection. Nine animal ACE2s showed intermediate susceptibility, ranging from 20 to 40% of WT S/hACE2 infectivity, including horse, alpaca, pig, squirrel, fox, raccoon dog, otter, and Crocuta. Chicken (10%), mouse (12%), rat (17%), and ferret (13%) ACE2s displayed less than 20% infectivity to hACE2, indicating weak or poor susceptibility. Guinea pig, tree shrew, hedgehog, koala, turtle, and zebrafish appeared non-susceptible to infection of all SARS-CoV-2 variants, displaying close to background level of infection to almost all SARS-CoV-2 variant S pseudoviruses tested (Figure 3B). Therefore, they were removed from further analysis.

Similar to WT, all omicron variants also exhibited very broad potential host range, but with some noticeable differences from WT (Figure 3B). There were five animal ACE2s, including squirrel, mouse, rat, fox, and civet, exhibiting 1.5-fold higher infectivity for pseudovirions of all omicron variants than WT. Specifically, transduction efficiencies for squirrel ACE2 were increased over WT by 1.5-fold, 2.4-fold, 3.5-fold, 2.4-fold, and 2.4-fold for BA.1, XBB.1.16, EG.5.1, BA.2.86, and JN.1, respectively. Similarly, transduction efficiencies of BA.1, XBB.1.16, EG.5.1, BA.2.86, and JN.1 pseudovirions were increased over WT by 3.5-fold, 5.7-fold, 7.4-fold, 5.5-fold, and 7.4-fold for mouse ACE2, by 2.3-fold, 3.9-fold, 4.7-fold, 2.8-fold, and 2.2-fold for rat ACE2, by 1.6-fold, 3.1-fold, 3.2-fold, 2.1-fold, and 1.9-fold for fox ACE2, and by 22.3-fold, 16-fold, 41-fold, 30-fold, and 18-fold for civet ACE2, respectively. Usage of chicken ACE2 by BA.1, XBB.1.16, EG.5.1 and BA.2.86 also showed more than 1.8-fold higher than WT, whereas JN.1 only exhibited background level of infectivity on chicken ACE2 (Figure 3B).

In contrast, pangolin ACE2s exhibited more than 66% reduction in infectivity by all omicron variants compared to WT S (Figure 3B). Specifically, transduction efficiencies on pangolin ACE2 by BA.1, XBB.1.16, EG.5.1, BA.2.86, and JN.1 S pseudoviruses were decreased by 99%, 70%, 66%, 71%, and 79%, respectively. Compared to WT S, close to 50% reduction in transduction efficiency was also shown on otter ACE2 by all omicron variants except for JN.1. Cat ACE2 also exhibited marked reduction in transduction efficiency for all omicron variants compared to WT, ranging from 34% for JN.1 to 77% for XBB.1.16. The rest of animal ACE2s only showed moderate differences between omicron variants and WT. Together, the marked increase of transduction efficiency mediated by squirrel, mouse, rat, fox and civet suggests they might be more susceptible to omicron variants than WT, while pangolins appear to be less susceptible. Of note, overall, JN.1 S pseudovirions exhibited significantly higher susceptibility to animal ACE2s than other SARS-CoV-2 variants tested, except for EG.5.1 (Figures 3B and 3C), indicating that the risk of reverse zoonotic transmission appears to arise as omicron variants are evolving.

### JN.1 S protein displayed significantly lower binding to various animal ACE2 orthologs than that of BA.2.86

**Figure 4:**
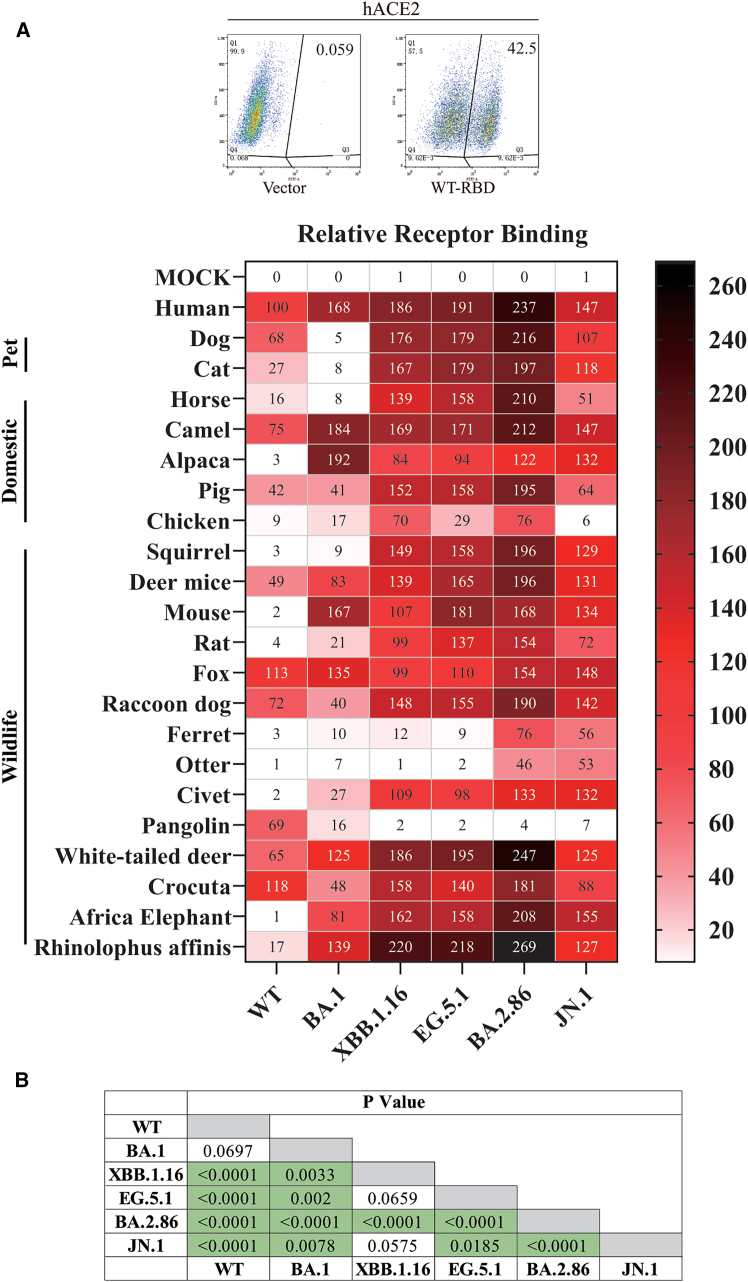
Receptor binding of omicron variants to various animal ACE2s (A) Relative receptor binding efficiencies of RBDs of WT, BA.1, XBB.1.16, EG.5.1, BA.2.86, and JN.1 variants to various animal ACE2s. HEK293T transiently expressing different animal ACE2s were detached with EDTA and incubated with mouse Fc-tagged RBDs of WT, BA.1, XBB.1.16, EG.5.1, BA.2.86, or JN.1, followed by FITC-conjugated polyclonal goat anti-mouse antibodies. The cells were analyzed by flow cytometry. Receptor binding efficiencies were calculated according to WT/hACE2, set as 100%. Experiments were performed three times, and the averages are shown in the heatmap. (B) Statistic analyses of levels of receptor binding between different omicron variant S protein by Wilcoxon test. Significant differences indicated by *p* < 0.05 are highlighted in green background.

To elucidate the underlying mechanism for shifts in ACE2 usage of omicron variants, we then examined the binding of these ACE2s to RBDs of various omicron variants using flow cytometry, since receptor binding is the first step for virus entry. HEK293T cells transiently expressing different animal ACE2 orthologs were incubated with individual soluble mouse Fc-tagged omicron RBDs, and binding was quantified using FITC-conjugated polyclonal anti-mouse antibodies. The relative binding is presented as the percentage of WT RBD binding to hACE2 (WT RBD/hACE2) (Figure 4A). WT RBD exhibited relative strong binding to ACE2s of dog, camel, fox, raccoon dog, pangolin, white-tailed deer, and Crocuta at levels of over 65% of hACE2 (Figure 4A), consistent with previous reports.^26^^,^^27^^,^^52^ ACE2s of cat, horse, pig, deer mice, and *Rhinolophus affinis* bat also displayed moderate binding to WT RBD, ranging from 16% to 49% of hACE2. ACE2s of alpaca, chicken, squirrel, mouse, rat, ferret, otter, and civet showed close to background levels of binding, consistent with their relative lower transduction efficiency. Strikingly, despite that ACE2 of African elephant almost showed 60% transduction efficiency of hACE2 to WT S pseudovirions, it only displayed background level of receptor binding activity to WT RBD (Figure 4A).

Compared to WT RBD, overall affinities of omicron RBDs with different animal ACE2s were markedly increased, with XBB.1.16, EG.5.1, BA.2.86, and JN.1 exhibiting significant higher affinities (Figures 4A and 4B). The patterns of receptor binding for XBB.1.16, EG.5.1, BA.2.86, and JN.1 are very similar among themselves but markedly different from WT and BA.1. Sixteen out of twenty-one animal ACE2 orthologs exhibited marked increase in binding to RBDs of XBB.1.16, EG.5.1, BA.2.86, and JN.1, compared to WT RBD, including ACE2s of squirrel, mouse, rat, ferret, and civet displaying more than 3-fold increase in receptor binding, consistent with their marked increase in susceptibility. In contrast, RBDs of all omicron variants bound to pangolin ACE2 poorly, in agreement with their low susceptibility for pangolin ACE2. Strikingly, while both Fox ACE2 and Crocuta ACE2s bound to all SARS-CoV-2 variants strongly (Figure 4A), Fox ACE2 exhibited significantly higher susceptibility than Crocuta ACE2 (Figure 3B), indicating that factors other than receptor binding might also be critical for virus entry. S proteins of BA.2.86 and JN.1 differ by a single amino acid, L455S, but JN.1 showed marked lower affinity with most animal ACE2s tested than BA.2.86, especially for chicken ACE2, indicating that the residue 455 might be important in interaction between ACE2s and S protein. Overall, BA.2.86 exhibited significant higher affinities with animal ACE2s than all other variants including WT (Figures 4A and 4B).

BA.1 showed unique binding pattern, with significant lower affinity than other omicron variants tested (Figure 4A). Specifically, BA.1 RBD poorly bound to ACE2s of dog, cat, horse, squirrel, ferret, otter, and pangolin, while it exhibited strong binding to ACE2s of camel, alpaca, deer mice, mouse, white-tailed deer, African elephant, and *Rhinolophus affinis* bat than WT. BA.1 RBD also displayed substantially higher affinity with ACE2s of chicken, rat, and civet than WT RBD with an increase of 1.9-fold, 5.3-fold, and 13.5-fold, respectively, consistent with their higher susceptibility than WT S pseudoviruses.

### JN.1 S protein induced substantially higher syncytium formation with animal ACE2 orthologs than BA.2.86

**Figure 5:**
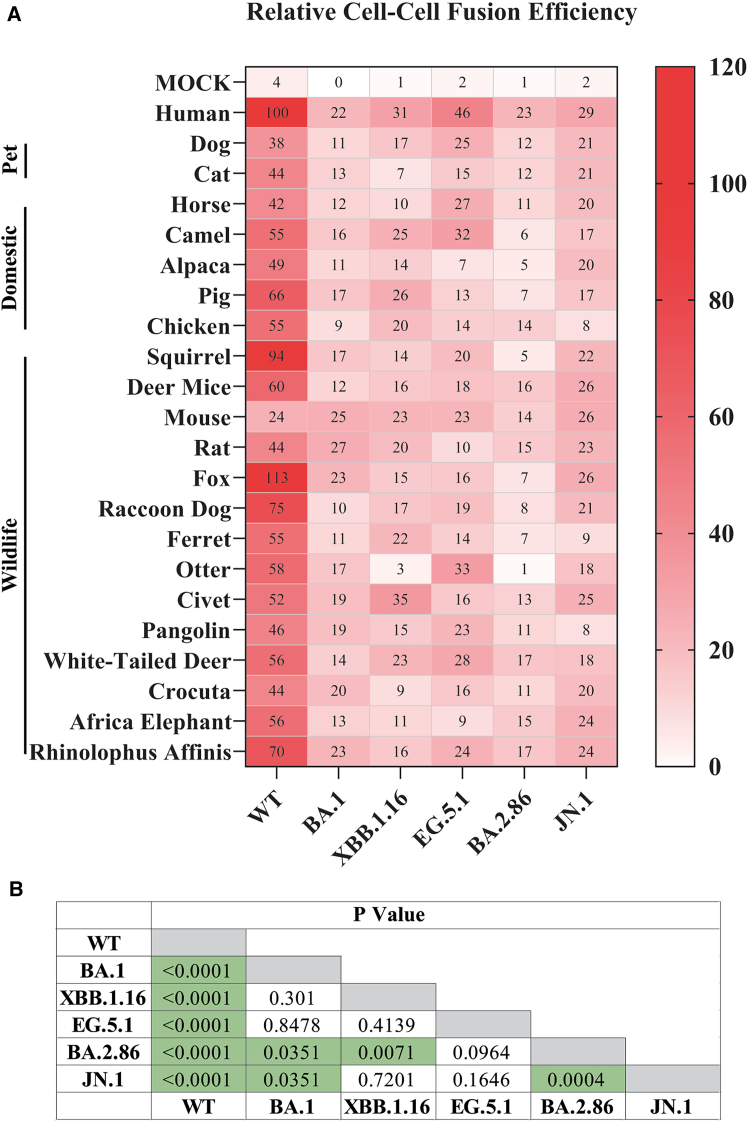
Cell-cell fusion mediated by different omicron variant S proteins on HEK 293 cells expressing various animal ACE2s (A) Relative fusion efficiencies of WT, BA.1, XBB.1.16, EG.5.1, BA.2.86, and JN.1 on HEK 293 cells expressing animal ACE2 orthologs. HEK 293T cells transiently co-expressing S protein and eGFP were detached with trypsin and overlaid on HEK 293 cells expressing different animal ACE2s. After 2 h of incubation, the images of syncytia were captured using a fluorescent microscope. Fusion areas were quantified using ImageJ software and normalized to WT/hACE2, set as 100%. Experiments were performed three times, and the averages are shown in the heatmap. (B) Statistic analyses of levels of cell-cell fusion between different omicron variant S proteins by Wilcoxon test. Significant differences indicated by *p* < 0.05 are highlighted in green background.

Following the receptor binding, viral and cellular membrane fusion represents another critical step for the virus to achieve successful entry. Additionally, cell-cell fusion also plays a critical role in SARS-CoV-2 pathogenesis.^53^ We previously showed that the WT S protein induced marked syncytia with cells transiently expressing various animal ACE2 orthologs.^28^ To evaluate the efficiency of syncytium formation mediated by animal ACE2 orthologs and the S protein from different omicron variants, HEK293T cells transiently co-expressing the S protein and eGFP were overlaid onto HEK293 cells also transiently expressing ACE2 orthologs. Fusion efficiency was also calculated as the percentage of fusion of WT S/hACE2 (set as 100%). As shown in Figure 5A, WT S mediated various levels of syncytia with animal ACE2 orthologs, ranging from 24% for mouse ACE2 to 113% for fox ACE2s, with most around 40–70%. Strikingly, in spite of ACE2s of alpaca, chicken, squirrel, mouse, rat, ferret, otter, civet, Africa elephant only exhibiting background level of receptor binding for WT RBD, they induced substantial levels of syncytia with WT S expressing cells (Figure 5A), in agreement with moderate levels of pseudovirus transduction mediated by these ACE2s. Compared to WT, all five omicron variants tested showed overall significantly attenuated membrane fusion capability with animal ACE2 orthologs (*p* < 0.0001) (Figure 5B), of which BA.2.86 appears to be the worst (*p* < 0.003) (Figure 5B). BA.1, XBB.1.16, and EG.5.1 exhibited no significant differences in overall syncytium-inducing patterns among themselves, and JN.1 displayed stronger fusogenicity than BA.1 (*p* = 0.0054) and BA.2.86 (Figures 5A and 5B).

### Comparative infectivity of JN.1 and BA.2.86 live viruses across various ACE2 orthologs

**Figure 6:**
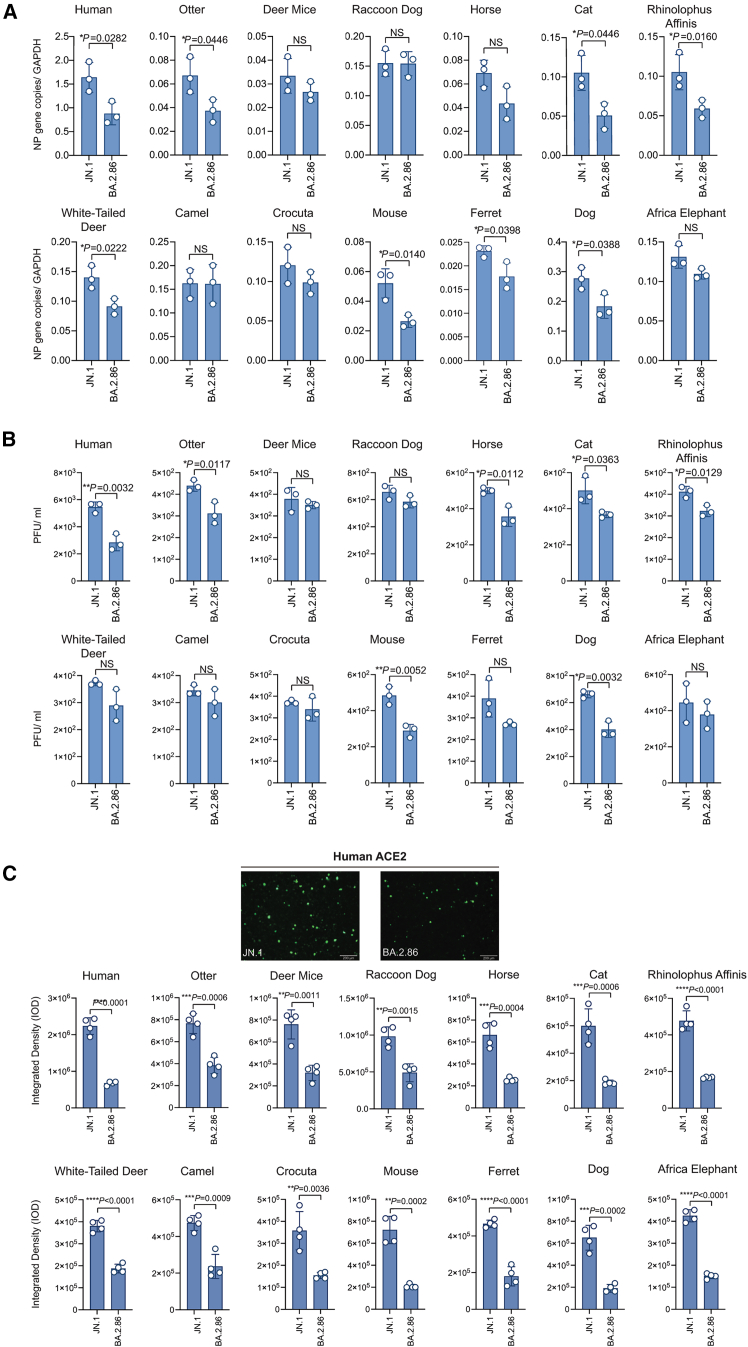
Comparison of JN.1 and BA.2.86 live virus infection on HEK293 cells expressing various ACE2 orthologs (A) Viral NP gene copies at 24 h post infection were quantified by RT-qPCR. (B) Viral titers at 24 h post infection were determined by plaque assay. (C) Viral infection induced syncytium formation. HEK293T cells were either co-transfected with the indicated ACE2 plasmid and GFP1-10 plasmid or transfected with GFP11 plasmid. At 24 h transfection, cells were lifted, mixed at a ratio of 1:1, and cultured in an 8 well chamber slides for 24 h. The mixed cells were infected with either JN.1 or BA.2.86 at multiplicity of infection (MOI) at 1.5 for 24 h and fixed with 4% paraformaldehyde. Images were captured using the Olympus BX73 fluorescence microscope and quantified using ImageJ. Experiments were done twice in triplicate, and one representative was shown as mean ± SD. Statistical significance was calculated using Student’s t test. (∗*p* < 0.05, ∗∗*p* < 0.01, ∗∗∗*p* < 0.001, ∗∗∗∗*p* < 0.0001, ns = not significant). Scale bar = 200μm. See also Figure S3.

To further assess whether the JN.1 variant has higher reverse zoonotic transmission risk than other Omicron variants, we compared the infectivity of authentic JN.1 and BA.2.86 live viruses across 14 different ACE2 orthologs (13 animal ACE2s and hACE2), measured viral RNA levels using real-time quantitative PCR (Figure 6A), and determined viral titers via plaque assay (Figure 6B). Among the 14 ACE2 orthologs tested, 8 showed significantly higher viral RNA production by JN.1 than BA.2.86, 4 exhibited a higher viral RNA level with JN.1 that did not reach statistical significance, and 2 displayed nearly identical RNA levels. Similarly, viral titers were higher with JN.1 in 7 ACE2 orthologs, while the other 7 showed a higher, yet not statistically significant, titer with JN.1. These findings largely align with the pseudoviral infection data (Figure 3), indicating that JN.1 may present a greater risk for reverse zoonotic transmission than BA.2.86.

We also compared levels of cell-cell fusion mediated by JN.1 and BA.2.86 virus infection using a split-GFP cell-cell fusion assay, in which two split-GFP fragments were expressed in two different cells and only these two types of cells fused to generate GFP. JN.1 virus exhibited significantly higher fusion efficiency when compared to BA.2.86 at 24 h post infection across all tested animal ACE2 orthologs (Figure 6C and Figure S3), in agreement with S protein mediated cell-cell fusion (Figure 5), indicating that JN.1 virus may be more fusogenic than BA.2.86.

### L455S mutation renders increase in infectivity and fusogenicity of S protein through lowering the stability of S protein in prefusion conformation

**Figure 7:**
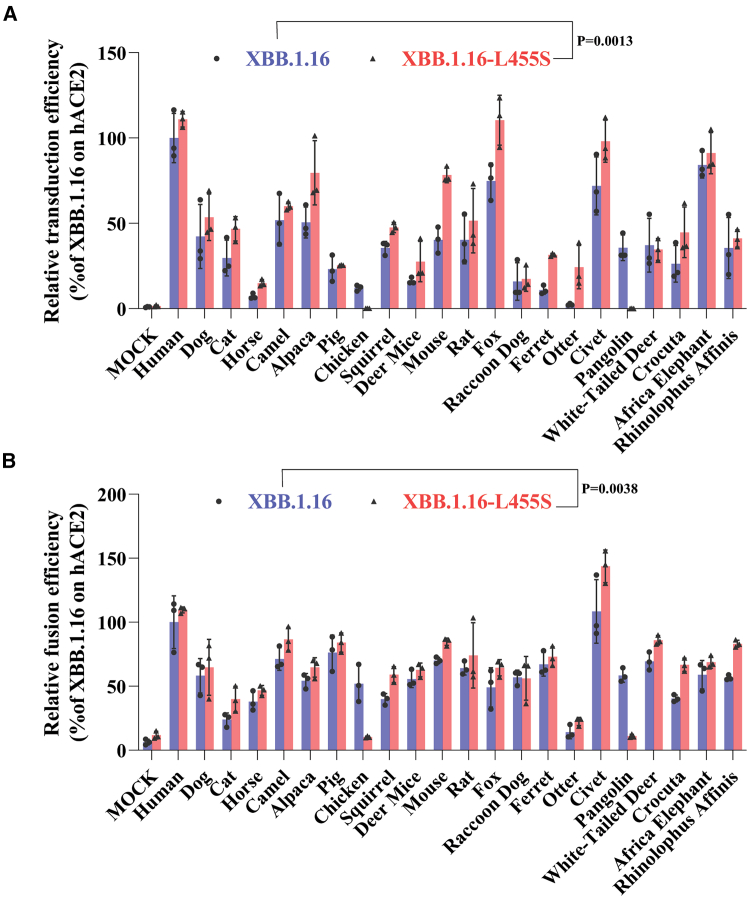
Effect of L455S mutation on transduction efficiency and fusogenicity of XBB.1.16 S protein (A) Relative transduction efficiencies of XBB.1.16 and XBB.1.16-L455S on HEK 293 cells expressing different animal ACE2s. HEK 293 cells transiently expressing different animal ACE2s were transduced by either XBB.1.16 WT S or L455S mutant S pseudovirions. The luciferase activities were measured at 40 h post transduction. Relative transduction efficiencies were calculated based on WT/hACE2(100%). Experiments were performed three times in triplicates and the representative one was shown. (B) Cell-cell fusion mediated by XBB.1.16 and XBB.1.16-L455S S proteins. HEK 293T cells transiently co-expressing S protein and eGFP were detached with trypsin and overlaid on HEK 293 cells expressing different animal ACE2s. After 2 h of incubation, the five randomly selected images of syncytia were captured using a fluorescent microscope. Fusion areas were quantified using ImageJ software and normalized to WT/hACE2, set as 100%. Experiments were performed twice and the representative one was shown. Statistical analyses were done by Wilcoxon test. See also Figure S4.

Given that S protein of JN.1 differs from that of BA.2.86 only by a single L455S mutation and exhibits significantly higher fusogenicity and infectivity than BA.2.86 with various animal ACE2s, we then determined whether the effect of L455S on S protein fusogenicity and infectivity is general or not. L455S mutation was introduced into S protein of XBB.1.16 variant, and the effect on transduction and fusogenicity of S protein with various animal ACE2s was measured. XBB.1.16 S proteins with L455S mutation (XBB.1.16-L455S) were expressed and incorporated into pseudovirions as well as WT XBB.1.16 (Figure S4). Among 22 ACE2 tested, 19 ACE2s including hACE2 exhibited increase in infectivity by L455S mutant S over WT XBB.1.16, whereas only three animal ACE2s including chicken, pangolin, and white-tailed deer showed reduction in transduction efficiency by L455S substitution (Figure 7A). Wilcoxon test revealed that L455S mutation in XBB.1.16 background displayed significant increase in infectivity with various animal ACE2 orthologs over WT XBB.1.16 (*p* = 0.0013), in agreement with the notion that L455S substitution might increase viral infectivity in general. We also evaluated the effect of L455S mutation on syncytium formation mediated by S proteins (Figure 7B). L455S mutant also exhibited significantly higher syncytium formation than WT XBB.1.16 among various animal ACE2s tested (*p* = 0.0038), except for ACE2s of chicken and pangolin, indicating that increase in infectivity by L455S mutation might result from augment of the fusogenicity. Of note, similar to JN.1, XBB.1.16 S pseudovirions with L455S mutation also gave close to the background level of infectivity in chicken ACE2 (Figure 7B), suggesting that residue 455 in S protein might be critical for interaction with chicken ACE2.

**Figure 8:**
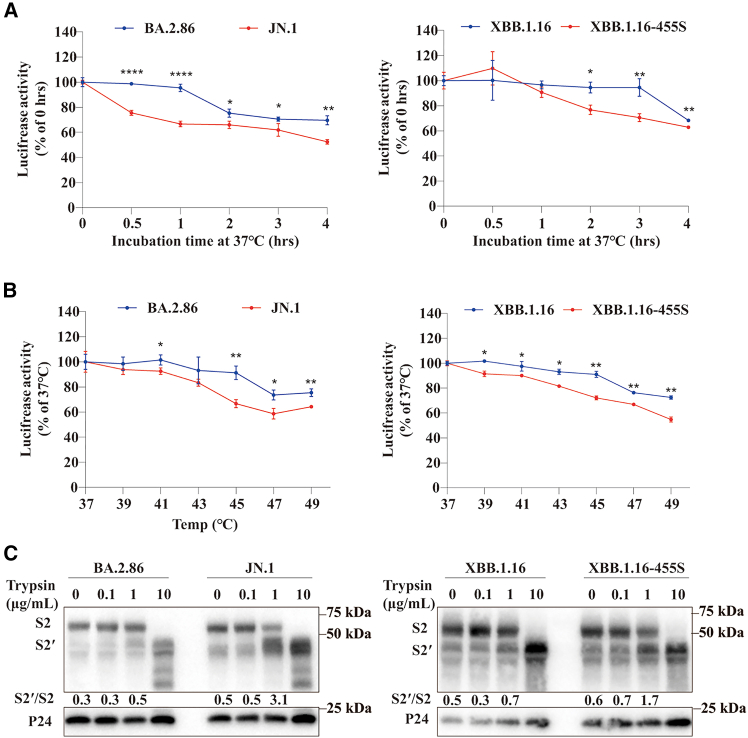
Effect of L455S mutation on thermal and proteolytic stability on XBB.1.16 and BA.2.86 (A and B) Thermal stability of JN.1 compared to its parental BA.2.86 (left panel) and XBB.1.16–455S to XBB.1.16 (right panel). Pseudovirus of XBB.1.16, XBB.1.16–455S, BA.2.86 and JN.1 were centrifuged through 20% sucrose to remove serum and resuspended in serum-free DMEM, followed by incubation either at 37°C for the indicated time (A) or at the indicated temperature for 2 h (B). The virus suspension was used to transduce 293/hACE2 cells to access their remaining transduction capability. Experiments were performed three times in triplicate, and the representative one was shown. The statistical difference was determined by a multiple t-test (*n* = 3). Error bars indicate SEM. ∗*p* < 0.05, ∗∗*p* < 0.01, ∗∗∗*p* < 0.001, ∗∗∗∗*p* < 0.0001. (C) Proteolytic stability of JN.1 compared to its parental BA.2.86 (left panel) and XBB.1.16–455S to XBB.1.16 (right panel). Pseudovirus of XBB.1.16, XBB.1.16–455S, BA.2.86 and JN.1 were resuspended in serum-free DMEM and incubated with soluble hACE2(20 μg) at 37°C for 30 min, followed by incubation with TPCK-treated trypsin. The mixture was immediately boiled with loading buffer containing DTT and separated in a 10% SDS-PAGE. Detection was carried out using rabbit polyclonal anti-SARS-CoV-2 S2 antibodies (1:3000). The numbers below S protein blot are the relative quantification of ratio of S2’/S2 using Image Lab (Bio-Rad). Experiments were performed at least three times, and the representative one was shown.

Previous studies in glycoproteins of coronavirus, ebolavirus, and other enveloped viruses showed that individual mutation might have substantial impact on thermostability of glycoprotein and viral fitness through lowering the threshold of energy barrier for conformational changes of glycoprotein that mediate membrane fusion.^54^^,^^55^^,^^56^^,^^57^ We then investigated whether L455S substitution has any effect on thermostability of S proteins. Pseudovirons containing S proteins were incubated at a specific temperature between 37°C and 49°C for the indicated time (Figure 8), and the remaining infectivity was determined. Compared to BA.2.86 and XBB.1.16, pseudoviral particles containing L455S mutation, JN.1 and XBB.1.16-L455S, were substantially more sensitive to thermal inactivation (Figure 8A and 8B) and more readily triggered for conformational changes upon receptor binding, indicated by the trypsin digestion patterns (Figure 8C). JN.1 and XBB.1.16-L455S S pseudovirions yielded substantially higher S2’/S2 ratio than BA.2.86 and XBB.1.16 at 1 μg/mL of trypsin, respectively, upon receptor binding. Taken together, these findings show that L455S mutation reduces the stability of the prefusion conformation of S protein, and thus reduces the threshold for the conformational change upon receptor binding.

### L455S mutation augments the ability of immune evasion by SARS-CoV-2

**Figure 9:**
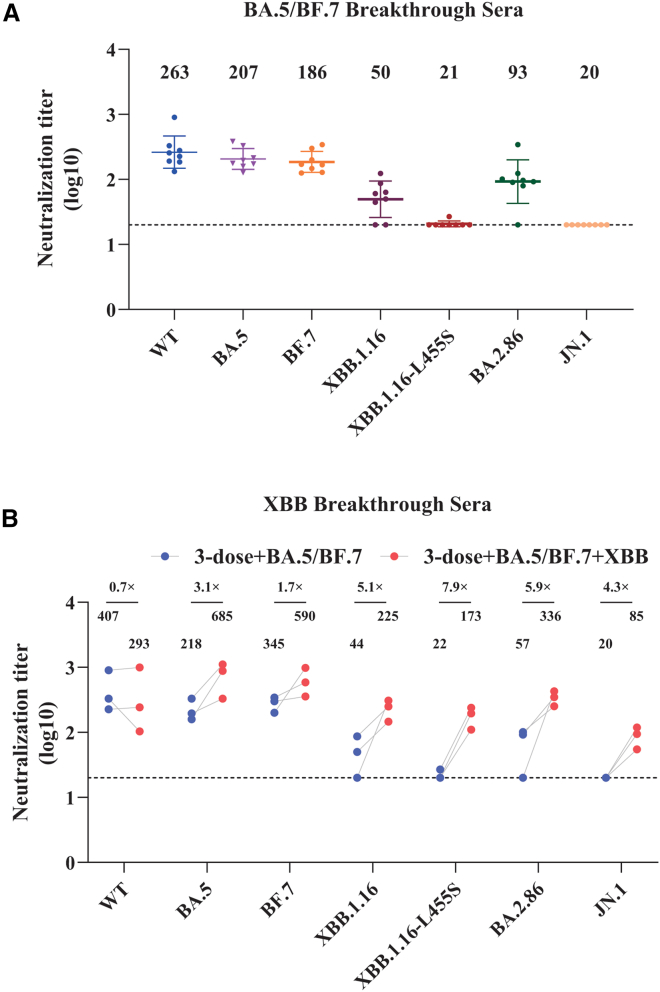
Cross-neutralization of BA.2.86 and JN.1 by sera from individuals with BA.5/BF.7 and XBB breakthrough infection (A and B) The NT_50_ of sera against WT, BA.5, BF.7, XBB.1.16, XBB.1.16-L455S, BA.2.86, and JN.1 were determined using pseudovirus-based neutralization assay. Sera were collected from individuals with three doses of inactivated SARS-CoV-2 vaccine and 14 days after recovery fromBF.5/BA.7 breakthrough infection alone (*n* = 8) (A) and from both BA.5/BF.7 and XBB breakthrough infection (*n* = 3) (B). Dashed lines represent the threshold of detection. Error bars indicate SEM.

Following the relaxation of stringent COVID-19 restrictions, about 85% of the Chinese population was infected during the BA.5/BF.7 wave between December 2022 to January 2023,^58^ followed by XBB outbreak from April to June, 2024 with about 61% of the population infected.^59^ To access levels of antibody protection against BA.2.86 and JN.1 infection, we obtained sera samples from a very small cohort including 5 individuals recovered from BF.5/BA.7 infection alone and 3 individuals recovered from both BA.5/BF.7 and XBB infection. All people have been immunized with 3 doses of inactivated SARS-CoV-2 vaccine prior to BA.5/BF.7 infection. Sera from individuals with BA.5/BF.7 breakthrough infection alone confers substantial level of neutralization against WT, BA.5, BF.7, and BA.2.86 with the neutralizing geometric mean titers (GMT) at 263, 207, 186, and 93 (Figure 9A), respectively, limited neutralization against XBB.1.16 with GMT at 50, and almost background level of neutralization activity against XBB.1.16-L455S and JN.1 (Figure 9A), indicating that L455S might be critical for immune evasion. Strikingly, additional XBB breakthrough infection significantly increased the neutralization titers against all omicron variants tested, including XBB.1.16-L455S and JN.1 (Figure 9B). Specifically, the GMTs for BA.5, BF.7, XBB.1.16, XBB.1.16-L455S, BA.2.86, and JNl.1 were increased by 3.1, 1.7, 5.1, 7.9, 5.9, and 4.3-fold (Figure 9B), respectively. Significant increase of GMTs against BA.2.86 and JN.1 by XBB infection indicates that addition of XBB into the vaccine component might provide considerable protection against BA.2.86 and JN.1. Of note, the GMT against WT S was slightly decreased even after XBB breakthrough infection, consistent with the ideal of immune imprint from initial vaccination with inactivated SARS-CoV-2 vaccine.

## Discussion

The COVID-19 pandemic has posed significant challenges to global public health and caused immense losses in human lives and the global economy. Given widespread human transmission of SARS-CoV-2 and its ability to infect a broad range of mammalian hosts, the establishment of new wildlife reservoirs of SARS-CoV-2 would present a new set of challenges, further complicate public health control measures, and could lead to some uncertainty of viral evolution and pathogenesis. Mink and white-tailed deer spillovers with extensive animal-to-animal transmission have been well documented,^35^^,^^36^^,^^37^^,^^60^^,^^61^ and some wild martens, badgers, and other animals were also seropositive for SARS-CoV-2.^62^ SARS-CoV-2 transmission from infected animals to humans was also proven by the phylogenetic analysis.^36^ As SARS-CoV-2 continuously evolves, omicron variants emerged with significant higher affinity with hACE2 than WT, particularly, newly emerged variants like BA.2.86 with more changes in RBD exhibiting even higher affinity with hACE2 (Figure 1D),^18^^,^^19^^,^^40^^,^^63^ raising the question of whether they might alter the animal host range and pose new potential reverse zoonotic transmission risk. Indeed, in this study, we found that JN.1 has significant higher zoonotic transmission potential than WT (Figures 3B, 6A, and 6B), and rodents and civet are more susceptible to omicron infection than WT (Figure 3B), indicating that additional surveillance of related animals might be needed.

There is no clinical evidence that JN.1 infection causes more severe diseases than any other XBB variants, but enhanced infectivity in nasal epithelial cells and increased fecal shedding has been documented in JN.1 patients,^64^^,^^65^ suggesting that JN.1 might have higher infectivity and cause more systemic infection.^66^ In this study, we also showed that JN.1 S pseudovirions displayed significantly higher infectivity in Calu3 cells than BA.2.86 (Figures 2B and 2C) and JN.1 live virus also exhibited substantial higher infectivity and fusogenecity than BA.2.86 across 14 different ACE2 orthologs tested, consistent with previous studies^47^^,^^65^ and supporting the idea that JN.1 exhibits increase in viral fitness over BA.2.86.^63^ Although BA.2.86 and JN.1 differ by only a single L455S substitution, they show significant differences in receptor binding,^40^^,^^63^^,^^67^ membrane fusion, and infectivity across various animal ACE2 receptors (Figures 3, 5, and 6). In silico analysis reveals that, compared to BA.2.86, JN.1 has reduced binding to most ACE2 orthologs likely due to its smaller side chain of S455, which results in loss of its van der Waals interactions with resides 30 and 34 of ACE2s. Despite this, overall affinities to various ACE2 receptors remain significantly higher than WT S (Figure 4A). In the case of weak binder like chicken ACE2, which has much smaller and hydrophobic side chains at A30 and V34, the L455S change has a more pronounced effect on receptor binding. In contrast, ACE2 receptors with larger and more hydrophilic side chains at residues 30 and 34, such as those of fox and civet, show that the hydrophilic S455 of JN.1 spike RBD functions comparably to the hydrophobic but larger L455 in BA.2.86. Of note, the effect of L455S mutation appears to be general, regardless of S protein backbone. Replacement of L455 in XBB.1.16 with serine displayed a similar effect on viral infectivity and fusogenicity to that of JN.1.

During 2013–2016 Ebola virus outbreak, two mutations, A82V and T544U, in glycoprotein (gp) were linked to higher infectivity and transmissibility of Ebola virus.^54^^,^^56^ Further study revealed that both mutations might reduce the thermostability of gp, resulting in decrease of the energy barrier required for conformational change of gp protein upon receptor binding.^57^ We hypothesized that L455S mutation might also affect the thermostability of the S protein. Indeed, we found that, compared to BA.2.86 and XBB.1.16, pseudovirions with L455S mutation were less stable (Figures 8A and 8B) and their S proteins were more easily activated, as reflected by higher S2’/S ratio present in trypsin cleavage patterns (Figure 8C), supporting the notion that L455S mutation might augment viral infectivity and fitness through reducing the stability of the prefusion conformation of S protein.

L455S mutation also exhibits profound effect on immune evasion. Sera from individuals who had received three doses of inactivated SARS-CoV-2 vaccine and BA.5/BF.7 breakthrough infection displayed almost no neutralization activity against XBB.1.16-L455S and JN.1, but noticeable neutralization titers against XBB.1.16 and BA.2.86 with GMTs at 50 and 93, respectively, indicating the strong immune evasion by L455S mutation, in agreement with previous reports.^47^^,^^48^^,^^65^ Of note, XBB breakthrough infection significantly enhanced the neutralizing titers against all omicron variants including BA.2.86 and JN.1, although GMTs against BA.2.86 remained substantially higher than those against JN.1. These observations are in line with other recent reports.^18^^,^^19^^,^^40^^,^^63^^,^^68^ The marked increase in neutralization titers against BA.2.86, JN.1, and other omicron variants by XBB breakthrough infection suggests that it might be a good idea to include XBB S proteins in COVID-19 vaccine regimens.

Given that L455S has a significant selective advantage in viral infectivity and immune evasion, why is this mutation not selected out among earlier variants in circulation? We speculate that the combination of significant negative effect on receptor binding by L455S with status of populational immunity against SARS-CoV-2 might be two major causes. Substantial increase in receptor binding by BA.2.86 might be prerequisite for tolerance of negative effect of L455S in receptor binding, maintaining the receptor binding affinity above the threshold required for triggering the conformational changes of S protein for membrane fusion.

Considering that virus entry is only the first step of the virus life cycle, post-entry block could restrict virus infection and make the host non-permissive. This study only determined the susceptibility of XBB.1.16, EG.5.1, BA.2.86, and JN.1 across various animal species, whether it might be true for infection remains to be determined.

In conclusion, we determined the potential host ranges of several omicron variants among various animal species and found that JN.1 variant exhibited elevated reverse zoonotic risk potential. More importantly, we identified L455S mutation responsible for increase in fusogenicity and infectivity among different animal ACE2s.

### Limitations of the study

This study has several limitations. First, findings rely on *in vitro* pseudovirus and cell culture models, which may not fully reflect *in vivo* infection dynamics or interspecies transmission barriers. Second, while susceptibility was assessed via viral entry, post-entry restrictions (e.g., host immune responses or replication efficiency) were not examined. Finally, the animal ACE2 panel excludes some wildlife species, limiting insights into full host range risks.

## Resource availability

### Lead contact

Further information and requests for resources and reagents should be directed to and will be fulfilled by the lead contact, Zhaohui Qian (Email: zqian2013@sina.com).

### Materials availability

This study did not generate new reagents.

### Data and code availability

• Data: All study data are included in the article and/or supplemental information.
• Code: This paper does not report previously unpublished custom code.
• Any additional information required to reanalyze the data reported in this paper is available from the lead contact upon request.

## Acknowledgments

This work was supported by the National Key R&D Program of China (2023YFC2307800, 2023YFC3041500 and 2022YFE0210300), the National Natural Science Foundation of China (32270174 and 32370181), Beijing Natural Science Foundation (L222009 and 5222032), the CAMS Innovation Fund for Medical Sciences (2022-I2M-CoV19-002), the Open Fund from State Key Laboratory of Veterinary Public Health Security (2024SKLVPHS02).

## Author contributions

Z.Q. conceived the projects. Z.Q. and J.L. coordinated the projects. Z.Q., J.L., J.H., X.T., and X.O. designed the experiments. J.H., F.Z., Y.H. Y.L., Pei Li, X.L., S.D., Y.C., Z.M., L.T., and T.T.-T.Y. performed all the experiments. Z.Q., J.L., J.H., X.O., Pinghuang Liu, and Pei Li analyzed the data. Z.Q. and J.H. wrote the manuscript with input from all co-authors.

## Declaration of interests

The authors declare no competing interests.

## STAR★Methods

### Key resources table

**Table:** 

REAGENT or RESOURCE	SOURCE	IDENTIFIER
**Antibodies**		

Rabbit polyclonal against SARS-CoV-2 S2 antibodies	SinoBiological Inc (Beijing, China)	Cat#40509-T62
Rabbit polyclonal against HIV-1 Gag-p24 antibodies	SinoBiological Inc (Beijing, China)	Cat#11695-T62
Mouse polyclonal against FLAG-tag antibodies	SinoBiological Inc (Beijing, China)	Cat#109143-MM13
Mouse polyclonal against beta-actin antibodies	SinoBiological Inc (Beijing, China)	Cat#100166-MM10
Chimeric monoclonal against SARS-CoV-2 S2 antibody	SinoBiological Inc (Beijing, China)	Cat#40590-D001
Alexa Fluor 488-conjugated streptavidin	Bioss Biological Inc (Beijing, China)	Cat#bs-0437P-AF488
Goat anti-human polyclonal cy5-conjugated antibodies	Bioss Biological Inc (Beijing, China)	Cat#bs-0297G-Cy5
Goat anti-mouse polyclonal FITC-conjugated antibodies	Bioss Biological Inc (Beijing, China)	Cat#bs-0296G-FITC

**Bacterial and virus strains**		

SARS-CoV-2 BA.2.86 virus	Department of Microbiology, the University of Hong Kong (HKU https://doi.org/10.1038/s41467-024-53033-7	
SARS-CoV-2 JN.1 virus	Department of Microbiology, the University of Hong Kong https://doi.org/10.1038/s41467-024-53033-7	
DH5α Chemically Competent Cell	Tsingke Biotechnology Co.,Ltd.	TSC-C14

**Biological samples**		

Human sera	this paper	

**Chemicals, peptides, and recombinant proteins**		

RBD of SARS-CoV-2 WT(EPI_ISL_479681)	this paper	
RBD of SARS-CoV-2 BA.1(EPI_ISL_6640919)	this paper	
RBD of SARS-CoV-2 XBB.1.16(EPI_ISL_ 17619088)	this paper	
RBD of SARS-CoV-2 EG.5.1	this paper	
RBD of SARS-CoV-2 BA.2.86(EPI_ISL_18110065)	this paper	
RBD of SARS-CoV-2 JN.1	this paper	
Soluble hACE2	this paper	

**Critical commercial assays**		

QIAamp viral RNA mini kit	Qiagen	Cat. No. 52906
QuantiNova SYBR Green RT-PCR kit	Qiagen	Cat. No. 208152
Steady-Glo Reagent	Promega	E2520

**Experimental models: Cell lines**		

HEK293	ATCC	#CRL-1573
HEK293T	ATCC	#CRL-3216
Calu3	ATCC	#HTB-55
Expi293F	GIBCO	A14527
VeroE6		
VeroE6-TMPRSS2		

**Oligonucleotides**		

BA5-346T-F:GTGTTCAACGCGACCacaTTCGCCTCC GTGTACG		
BA5-346T-R:CGTACACGGAGGCGAAtgtGGTCGCG TTGAACAC		
K478R-F:GCTGGAAATagaCCTTGTAACGGCGTTGC CGGCC		
K478R-R:CAAGGtctATTTCCAGCTTGGTAGATCTCA GTAG		
F456L-F:TACCTGTACAGACTGctgAGAAAGAGCAA ACTG		
F456L-R:CAGTTTGCTCTTTCTcagCAGTCTGTACA GGTA		
RBD-F:CCCAAGCTTAACGTGACAAATCTGTGTC		
RBD-R:TCTGTACCAGTAGTCGTAGTTGCCAGAATG		

**Software and algorithms**		

SnapGene software	www.snapgene.com	
GraphPad Prism	www.graphpad.com	V9.5.1.733
NIS Element Br software	www.microscope.healthcare.nikon.com	
ImageJ software	https://imagej.net/ij/	V1.54f
ImageLab software	www.bio-rad.com	V6.1.0

### Experimental model and study participant details

#### Cell lines

HEK293 cells (ATCC #CRL-1573), HEK293T cells (ATCC #CRL-3216), and Calu3 (ATCC #HTB-55) were maintained in Dulbecco’s modified Eagle medium (DMEM, GIBCO, Grand Island, USA) supplemented with 10% fetal bovine serum (FBS, GIBCO) and 1% penicillin-streptomycin-fungizone (PSF, GIBCO) at 37°C with 5% CO_2_. Suspension cells Expi293F (GIBCO, A14527) were cultured in Expi293 expression medium (GIBCO, A14535101) with 5% CO_2_ and 125 rpm. All cell lines used in this study were authenticated by short tandem repeat (STR) profiling and have been tested negative for contamination with mycoplasma.

#### Viruses

SARS-CoV-2 JN.1 and BA.2.86 isolates have been described previously.^65^ Viruses were grown in VeroE6 cells stably expressing transmembrane serine protease 2 (TMPRSS2). The VeroE6-TMPRSS2 cells were infected by viruses at a multiplicity of infection (MOI) of 0.01 for 2 to 3 days to generate a working stock. The virus supernatant was clarified by centrifugation (500 g × 5 min) and filtered through a 0.45-μm filter. The supernatant was then aliquoted for storage at −80°C. Viral titers were measured by standard plaque assay using VeroE6-TMPRSS2 cells as previously described.^69^ *In vivo* and *in vitro* experiments with infectious SARS-CoV-2 were performed according to the approved standard operating procedures of the Biosafety Level 3 facility at the Department of Microbiology, HKU.

#### Constructs and plasmids

The codon-optimized SARS-CoV-2 S constructs of B.1.1 (D614G variant, EPI_ISL_479681), BA.1(EPI_ISL_6640919), BA.2.75(EPI_ISL_13471039), BA.5(EPI_ISL_12029894), XBB.1.5(EPI_ISL_16134259), XBB.1.16(EPI_ISL_ 17619088), BQ.1.1(EPI_ISL_15579783), CH.1.1(EPI_ISL_16907910), BA.2.86(EPI_ISL_18110065), JN.1(EPI_ISL_18363371) with 19 residues deletion at C-terminal were synthesized by GenScript (Nanjing, China), and cloned into pcDNA3.1(+) vector between *Hind* III and *Xba* I sites to generate S protein expression plasmids. The BF.7, XBB.1.16, and EG.5.1 S expressing constructs were generated through mutagenesis using BA.5, XBB.1.5, and XBB.1.5, respectively, as templates, and verified by sequencing. The DNA sequences encoding RBD proteins (residues 331–528) were inserted into pcDNA3.1(+) with an N-terminal human IgG light chain signal peptide and C-terminal mFc and twin-strep tags. The coding sequence for soluble human ACE2 was cloned into the pcDNA3.1(+) vector with an N-terminal human IgG light chain signal peptide and a C-terminal twin-strep tag. The plasmids encoding animal ACE2 orthologs and hACE2 were synthesized by Sango Biotech (Shanghai, China) and cloned into the p3×Flag-CMV14 vector between the *Hind* III and *BamH* I sites. The lentiviral packaging plasmid psPAX2 was obtained from Addgene (Cambridge, USA). The pLenti-GFP lentiviral reporter plasmid that contains encoding sequences of GFP, and firefly luciferase was generously gifted by Fang Li (Duke University). Nucleotide sequences were determined by DNA sequencing services (RuiBiotech), and the sequence data were analyzed by SnapGene software (www.snapgene.com).

#### Antibodies

Rabbit polyclonal against SARS-CoV-2 S2 antibodies (Cat#40509-T62), rabbit polyclonal against HIV-1 Gag-p24 antibodies (Cat#11695-T62), mouse polyclonal against FLAG tag antibodies (Cat#109143-MM13) and mouse polyclonal against beta-actin antibodies (Cat#100166-MM10) for western blotting and chimeric monoclonal against SARS-CoV-2 S2 antibody (Cat#40590-D001) used for flow cytometry was purchased from SinoBiological Inc (Beijing, China). Alexa Fluor 488-conjugated streptavidin (Cat#bs-0437P-AF488), goat anti-human polyclonal cy5-conjugated antibodies (Cat#bs-0297G-Cy5), and goat anti-mouse polyclonal FITC-conjugated antibodies (Cat#bs-0296G-FITC) were purchased from Bioss Biological Inc (Beijing, China).

#### Human serum samples

Serum samples were obtained from eight convalescent COVID-19 patients, all of whom (*n* = 8, male:female = 3:5; median age: 29 years old, range: 23–35) had received three doses of either the CoronaVac Sinovac Biotech or BBIBP-CorV Sinopharm inactivated vaccines and recovered from BA.5.2/BF.7 infection. Three of them (male:female = 1:2; median age: 27 years old, range: 26–29) have recovered from XBB infection after BA.5.2/BF.7 infection. Sera were inactivated at 56°C for 30 min and stored at −80°C until use. Prior to participation, all volunteers provided informed consent. The study protocol was reviewed and approved by the Ethics Review Board of the National Institute of Pathogen Biology, Chinese Academy of Medical Sciences & Peking Union Medical College (IPB-2023-01).

### Method details

#### Production and transduction of pseudovirions

Pseudovirions with different Omicron S proteins were produced as described previously.^10^ Briefly, HEK 293T was transfected with the plasmids encoding S protein, psPAX2, and pLenti-GFP at a molar ratio of 1:1:1 using PEI. After 40 h of incubation, the supernatant containing pseudovirions was collected, and centrifuged at 500g for 10 min to remove cell debris. The viral supernatant was then added to pre-seeded receptor cells at approximately 30–40% confluency. After overnight incubation at 37°C, fresh media were added. Transduced cells were lyzed at 40 h post-inoculation using Steady-Glo Reagent (Promega, Madison, WI). Luciferase activities, indicative of transduction efficiency, were quantified using a Modulus II Microplate Reader (Turner BioSystems, Sunnyvale, CA, USA). All experiments were done in triplicate and repeated at least three times.

#### Western blotting

To analyze S protein expression, HEK 293T cells transfected with plasmids encoding either WT, BA.1, BA.2.75, BA.5, BF.7, BQ.1.1, CH.1.1, XBB.1.5, XBB.1.16, EG.5.1, BA.2.86, or JN.1 were lysed at 40 h post-transfection with RIPA lysis buffer consisting of 20 mmol/L Tris-HCl (pH 7.5), 150 mmol/L NaCl, 1 mmol/L EDTA, 0.1% sodium dodecyl sulfate (SDS), 1% NP40, and a protease inhibitor cocktail. After 30 min of incubation on ice, the cell lysates were centrifuged at 12,000 × g for 10 min at 4°C to remove nuclei. To evaluate the levels of S proteins incorporation in pseudovirions, the viral supernatants were first centrifuged at 25,000 rpm for 2 h at 4°C through a 20% sucrose cushion using a Beckman SW41 rotor to pellet pseudovirions. Virion pellets were then resuspended in 30 μL of 1x loading buffer, boiled for 10 min, and separated on a 10% SDS-polyacrylamide gel electrophoresis (PAGE) gel. The samples were then transferred to nitrocellulose filter membranes. After blocking with 5% milk, the membranes were incubated with primary antibodies, followed by horseradish peroxidase (HRP)-conjugated secondary antibodies (1:5000), and visualized with Clarity Western ECL substrate (BioRad, Hercules, CA, USA). The detection of flag-tagged ACE2s is the same as described above. The dilutions of the primary antibodies for western blotting were 1:3000 for the SARS-CoV-2 S2 antibodies, 1:5000 for the beta-actin antibodies, 1:2000 for the HIV-1 Gag-p24 antibodies, 1:3000 for mouse anti-FLAG M2 antibody, and 1:10000 for corresponding second antibodies.

#### Cell surface protein biotinylation assay

To assess the surface expression levels of ACE2 from different species, FLAG-tagged ACE2-expressing cells at 80%–90% confluence were incubated on ice for 30 min with PBS containing 2.5 μg/mL EZ-linked Sulfo-NHS-LC-LC-biotin (Thermo-Pierce, Waltham, US) following a wash with ice-cold PBS. The reaction was then quenched with PBS supplemented with 100 mmol/L glycine, and the cells were lysed using RIPA buffer. Biotin-labeled proteins were pulled down by incubating the lysates with NeutrAvidin beads (Thermo-Pierce) overnight at 4°C. After three washes with RIPA buffer, the samples were resuspended in 30 μL of loading buffer, boiled for 10 min, and analyzed by Western blotting using an anti-FLAG M2 antibody (1:1000). Integrin-β1 served as a loading control.

#### Receptor binding assay

For receptor binding assay in Figure 1, HEK293T cells transfected with plasmids encoding either WT, BA.1, BA.2.75, BA.5, BF.7, BQ.1.1, CH.1.1, XBB.1.5, XBB.1.16, EG.5.1, BA.2.86, or JN.1 were suspended using PBS containing 1 mmol/L EDTA, then washed using wash buffer (PBS containing 2% FBS). About 2 × 10^5^ S protein overexpressing cells were then incubated with 100μL soluble biotinylated hACE2(5μg/mL, diluted in wash buffer) for 1 h on ice. After washing three times using wash buffer, the cells were incubated with Alexa Fluor 488-conjugated Streptavidin (Cat#bs-0437P-AF488) antibody (1:500 dilution), followed by three times washing. For the receptor binding assay in Figure 4, 293T cells transfected with plasmids encoding animal ACE2s were incubated with the indicated RBDs and then mouse polyclonal FITC-conjugated antibody. The cells were then fixed with 1% paraformaldehyde (PFA; Solarbio) and analyzed by BD LSRFortessa Cell Analyzer (BD Biosciences).

#### Cell-cell fusion assay

The 293T cells transiently expressing S protein and eGFP were detached with trypsin (0.25%, Gibco) and overlaid on top of a 70% confluent monolayer of cells expressing ACE2, with a ratio of about one S-protein expressing cell to every three receptor-expressing cells. After 4 h co-culture, images of syncytia were captured using a Nikon TE2000 epifluorescence microscope, equipped with NIS Element software (Nikon, Japan). Fusion area were quantified using ImageJ software.^70^ The relative fusion efficiency is defined as the ratio of fusion areas.

#### Virus-infection induced cell-cell fusion assay

Virus-infection induced cell-cell fusion assay was done as described previously.^71^ Briefly, HEK293T cells were either co-transfected with the indicated ACE2 plasmids and GFP1-10 plasmid (cat#68715, Addgene) or transfected with GFP11 plasmid (cat#68716, Addgene). After 24 h incubation, both cells were lifted with trypsin and mixed at a ratio of 1:1. The mixed cells were co-cultured in an 8-well chamber slides at 37°C for another 24 h. The cells were then inoculated with SARS-CoV-2 JN.1 or BA.2.86 at a MOI of 1.5. After 2 h of incubation, the virus inoculum was removed, cells were washed for three times with phosphate-buffered saline (PBS), and cells were fed with fresh DMEM supplemented with 1% sodium pyruvate and 2% FBS. After another 24 h incubation, the cells were fixed in 4% (wt/vol) paraformaldehyde at room temperature for 24 h. The antifade mounting medium with 4′,6-Diamidino-2-Phenylindole, Dihydrochloride (DAPI, H-1200, Vector Laboratories) was used for mounting and DAPI staining. Images were taken with the Olympus BX73 fluorescence microscope (Olympus Life Science, Tokyo, Japan). The fusion area of images was quantified by ImageJ.

#### Viral gene copy quantification by RT-qPCR

HEK293T cells transiently expressing the indicated ACE ortholog were inoculated with JN.1 or BA.2.86 at a MOI of 1.5. After 2 h incubation, viral inocula were removed and cells were washed three times with PBS and fed with fresh medium supplemented with 1% sodium pyruvate and 2% FBS. After 24 h incubation, viral supernatant was harvested and the viral NP gene copies were quantified as previously described.^72^ Briefly, about150 μL of viral supernatant were subjected for RNA extraction using QIAamp viral RNA mini kit (Qiagen, Hilden, Germany). Real-time quantitative RT-PCR analysis was carried out using QuantiNova SYBR Green RT-PCR kit (Qiagen) with a LightCycler 480 Real-Time PCR System (Roche, Basel, Switzerland).

#### Viral titration by plaque assay

The plaque assay was performed as described previously.^73^ Briefly, Vero E6-TMPRSS2 cells were maintained in DMEM with 10% FBS, 1% penicillin, and 1% streptomycin. The cells were seeded in 12-well plates at a density of 2 × 10^5^ cells per well. After overnight incubation, 10-fold serially diluted viral supernatant was added on VeroE6-TMPRSS2 cells. After 2 h incubation, the viral inoculum was removed, and the cells were washed with PBS for 3 times and overlaid with 1 mL of mixture with a ratio of 1:1 of 2% low-melting agarose and DMEM with 2% FBS and 2% P/S. After 4 days of incubation, the cells were fixed by 4% (wt/vol) paraformaldehyde for 24 h, and stained with 0.5% crystal violet in 25% ethanol for viral titer determination.

#### Expression and purification of SARS-CoV-2 RBDs and soluble hACE2

The Receptor Binding Domains (RBDs) from various strains and the soluble human ACE2 protein were produced within Expi293F cells (Gibco). About 1mg of the expression plasmids were transfected into 1×10^9^ 293F cells when the cell density reached 2×10^6^/mL using the Transfection Reagent (KHTEC, Beijing, China) according to manufacturer instructions. Cell culture supernatants were collected 3 days post-transfection and incubated with Strep-Tactin XT Superflow high-capacity resin beads (IBA Lifesciences) for 2 h at 4°C. The beads were then washed with wash buffer (20 mM Tris, 200 mM NaCl, pH = 8.0) before being eluted with wash buffer containing 50 mM biotin (Sigma). The eluates were concentrated using Amicon Ultra centrifugal filters with a 10 kDa molecular weight cutoff (Millipore, USA), aliquoted, and stored at −80°C.

#### Neutralization assay

The pseudovirions were mixed with human sera diluted in 2-fold serial dilutions (starting at 1:20) and incubated for 1 h at 37°C. The virus-antibody mixture was then added to 293/hACE2 cells in 96-well plates. Following an overnight incubation, the medium was replaced with fresh medium. The cells were lysed at 48 h post-transduction, and pseudovirus transduction was measured as described previously. NT_50_ was defined as previously described.^74^^,^^75^ All experiments were done in triplicate.

#### Trypsin digestion of S-pseudotyped lentiviruses

The supernatant containing pseudovirus was centrifuged through a 20% sucrose cushion at 25,000 rpm and 4°C for 2 h using a Beckman SW41 rotor. The resulting viral pellets were resuspended in PBS. The virus suspension was then incubated with 20 μg of soluble hACE2 at 37°C for 30 min to allow receptor binding and conformational change. The indicated amount of TPCK-treated trypsin was added, followed by a 30min of incubation at 37°C. After incubation, 5-fold excess of soybean trypsin inhibitors was added to stop the digestion. The mixture was immediately boiled with the loading buffer containing DTT and subjected to Western blot analysis using rabbit polyclonal anti-SARS-CoV-2 S2 antibodies (1:3000), followed by HRP-conjugated goat polyclonal against rabbit antibodies(1:10000).

#### Thermostability analysis of pseudotyped lentiviruses with S proteins

The supernatant containing pseudoviruses was centrifuged through a 20% sucrose cushion at 25,000 rpm and 4°C for 2 h using a Beckman SW41 rotor. The resulting viral pellets were resuspended in serum-free DMEM and incubated either at the indicated temperature for 2 h or at 37°C for the indicated time. The virus suspension was used to transduce 293/hACE2 cells to access their remaining infectivity.

### Quantification and statistical analysis

Data were analyzed for significance using one-way ANOVA with Dunnett’s multiple testing correction, two-tailed t-test or Wilcoxon test. ∗*p* < 0.05, ∗∗*p* < 0.01, ∗∗∗*p* < 0.001, ∗∗∗∗*p* < 0.0001, ns = not significant. Figure legends detail all quantification and statistical analyses, inclusive of numbers (n), and statistical tests. All statistical analyses were conducted using GraphPad Prism v9.5.1.733.
