# Supplementary material for: Enhanced reverse zoonotic potential and immune evasion by omicron JN.1 variant
Source: iScience. 2025 Jun 6;28(7):112824. doi: 10.1016/j.isci.2025.112824 (PMC12209934; doi:10.1016/j.isci.2025.112824)
Supplement: Document S1. Figures S1–S4 [file mmc1.pdf]

iScience, Volume *28*

Supplemental information

Enhanced reverse zoonotic potential and immune evasion by omicron JN.1 variant

Jiaxin Hu, Fuwen Zan, Yixin He, Xiuyuan Ou, Xiaolu Tang, Yan Liu, Xing Lu, Pei Li, Zhixia Mu, Siwen Dong, Yahan Chen, Lin Tan, Mengmeng Cao, Pinghuang Liu, Terrence Tsz-Tai Yuen, Jian Lu, and Zhaohui Qian

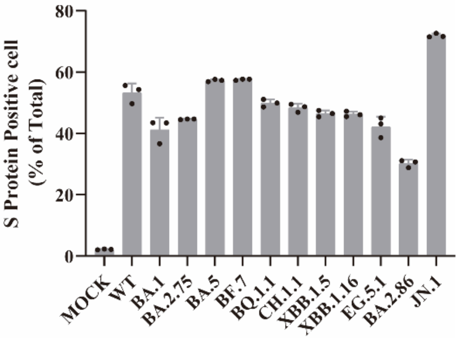

Figure S1. Expression of S proteins on cell surface, Related to Figure 1. Cells transiently expressing the indicated S proteins were incubated with chimeric monoclonal against SARS-CoV-2 S2 antibody followed by Cy5-conjugated goat anti-human polyclonal antibodies and analyzed by flow cytometry.

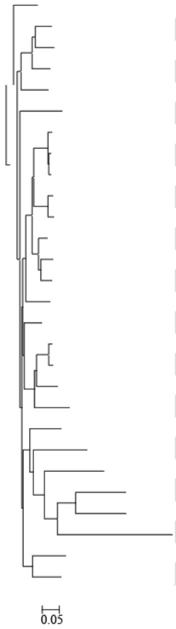

	24	27	28	30	31	34	35	37	38	41	42	79	82	83	330	353	354	355	357	393
Human	Q	T	F	D	K	H	E	E	D	Y	Q	L	M	Y	N	K	G	D	R	R
Mouse	N	-	-	N	N	Q	-	-	-	-	-	T	S	F	-	H	-	-	-	-
Rat	K	S	-	N	-	Q	-	-	-	-	-	I	N	F	-	H	-	-	-	-
Deer mice	-	I	-	-	-	Q	-	-	-	-	-	-	N	-	-	-	-	-	-	-
Squirrel	L	-	-	-	-	Q	-	-	-	H	-	-	D	-	-	-	-	-	-	-
Tree shrew	D	V	-	N	-	I	-	-	E	-	-	Q	R	-	K	N	N	-	-	-
Dog	L	-	-	E	-	Y	-	-	E	-	-	-	T	-	-	-	-	-	-	-
Fox	L	-	-	E	-	Y	-	-	E	-	-	-	T	-	-	-	-	-	-	-
Raccoon dog	L	-	-	E	-	Y	-	-	E	-	-	-	T	-	-	R	-	-	-	-
Ferret	L	-	-	E	-	Y	-	-	E	-	-	H	T	-	-	-	R	-	-	-
Otter	L	-	-	E	-	Y	-	-	E	-	-	N	T	-	-	-	R	-	-	-
Cat	L	-	-	E	-	-	-	-	E	-	-	-	T	-	-	-	-	-	-	-
Civet	L	-	-	E	T	Y	-	Q	E	-	-	-	T	-	-	-	-	-	-	-
Crocuta	L	-	-	E	-	Y	-	Q	E	-	L	-	T	-	-	-	-	-	-	K
Pangolin	E	-	-	E	-	S	-	-	E	-	-	I	N	-	-	-	H	-	-	-
Horse	L	-	-	E	-	S	-	-	E	H	-	-	T	-	-	-	-	-	-	-
Camel	L	-	-	E	E	-	-	-	-	-	-	T	T	-	-	-	-	-	-	-
Alpaca	L	-	-	K	E	-	-	-	-	-	-	A	I	-	-	-	-	-	-	-
Pig	L	-	-	E	-	L	-	-	-	-	-	I	T	-	-	-	-	-	-	-
White-tailed deer	-	-	-	E	-	-	-	-	-	-	-	M	T	-	-	-	-	-	-	-
African elephant	L	-	-	-	T	Q	-	-	-	-	-	-	D	F	-	-	-	-	-	-
Hedgehog	-	S	-	T	T	N	-	-	N	-	-	-	K	F	K	L	N	-	-	-
Koala	R	E	-	E	T	K	-	-	E	-	-	I	T	F	-	-	-	-	-	-
Chicken	-	-	-	A	E	V	R	-	-	-	E	N	R	F	-	-	N	-	-	-
Turtle	-	A	-	S	E	V	Q	-	-	-	A	N	K	-	-	-	K	-	-	-
Zebrafish	-	E	-	-	R	K	-	S	-	-	-	E	K	-	D	R	K	-	-	-
Guinea pig	L	I	-	-	E	S	-	-	N	-	-	-	N	-	-	-	N	-	-	-
Rhinolophus affinis	R	I	-	-	N	-	-	-	-	-	-	-	N	-	-	-	-	-	-	-

Figure S2. Phylogenetic analysis of animals based on ACE2 and alignment of S protein-contacting residues, Related to Figure 3. Phylogenetic tree based on ACE2 amino acid sequences was constructed using MEGA X. The 20 critical residues of hACE2 involved in interactions with the SARS-CoV-2 RBD are listed. Dashed lines represent residues identical to human ACE2.

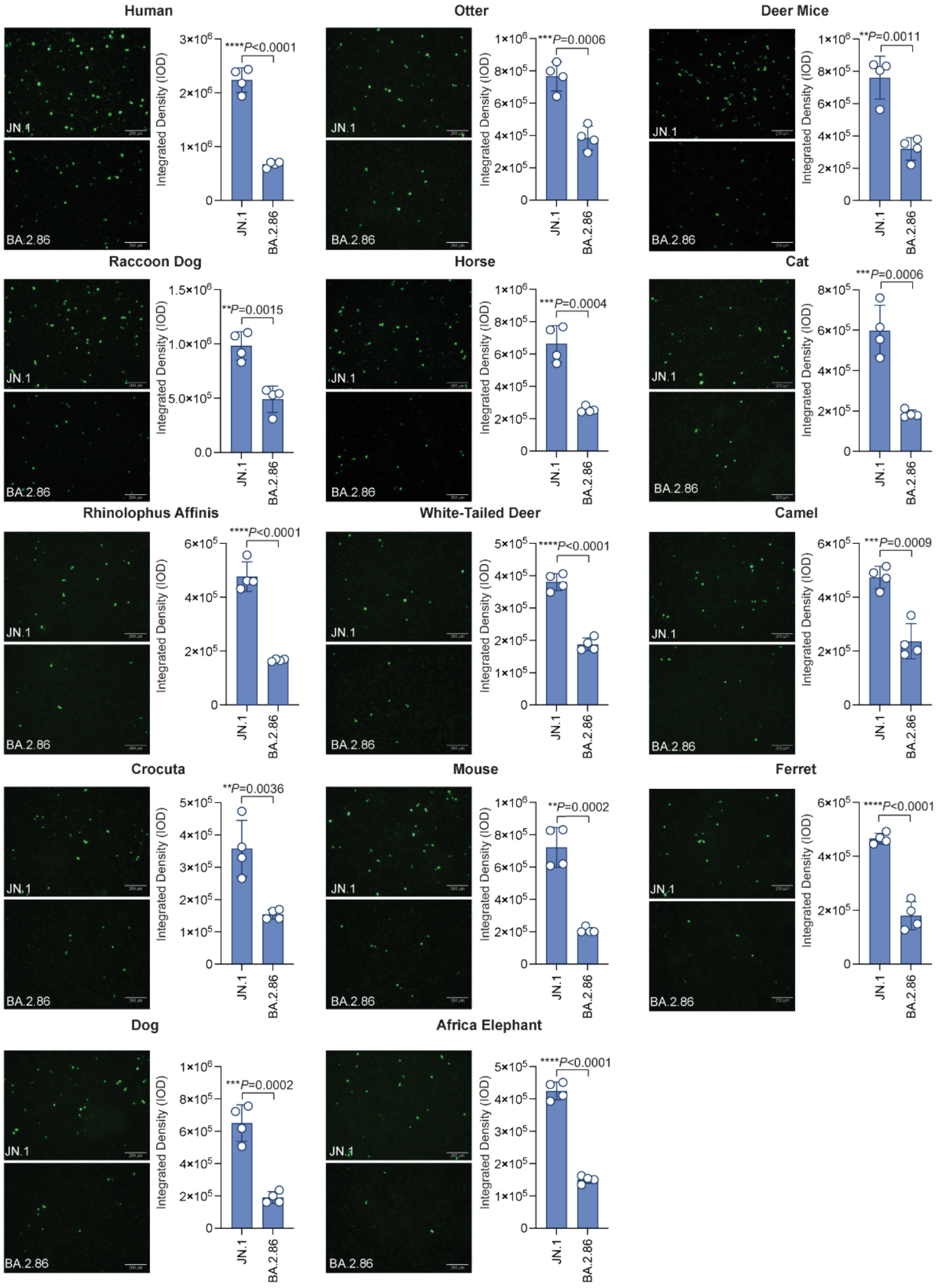

Figure S3. Viral infection induced syncytium formation, Related to Figure 6 Viral infection induced syncytium formation. HEK293T cells were either co-transfected with the indicated ACE2 plasmid and GFP1-10 plasmid or transfected with GFP11 plasmid. At 24 h transfection, cells were lifted, mixed at a ratio of 1:1, and cultured in an 8 well chamber slides for 24 h. The mixed cells were infected with either JN.1 or BA.2.86 at multiplicity of infection (MOI) at 1.5 for 24 hrs and fixed with 4% paraformaldehyde. Images were captured using the Olympus BX73 fluorescence microscope and quantified using ImageJ. Experiments were done twice in triplicate, and one representative was shown as mean ± SD. Statistical significance was calculated using Student's t-test. (*p < 0.05, **p < 0.01, ***p<0.001, ****p<0.0001, ns=not significant).

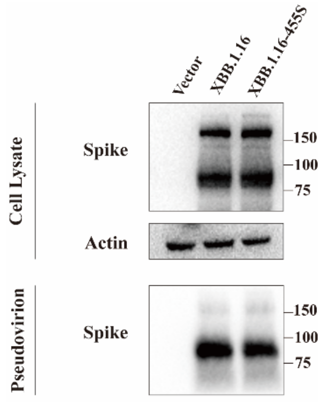

Figure S4. Detection of the S proteins of XBB.1.16 and XBB.1.16-L455S in cell lysate and pseudovirions by western blot, Related to Figure 7. Plasmids encoding XBB.1.16 or XBB.1.16-L455S S proteins were transfected into HEK293T cells and the cells were lysed at 40 hrs post-transfection. Pseudovirions were pelleted down by centrifugation through 20% sucrose cushion. Samples were separated in a 10% SDS-PAGE and the S proteins were detected by western blot using rabbit polyclonal anti-S2 antibodies. Actin and p24 served as the loading control.
